# Editorial: Special Issue on “Molecular Mechanisms Regulating Osteoclastogenesis”

**DOI:** 10.3390/ijms21207643

**Published:** 2020-10-15

**Authors:** Giacomina Brunetti, Giorgio Mori, Maria Felicia Faienza

**Affiliations:** 1 Department of Biosciences, Biotechnologies and Biopharmaceutics, University of Bari Aldo Moro, 70124 Bari, Italy; 2 Department of Clinical and Experimental Medicine, University of Foggia Medical School, 71122 Foggia, Italy; giorgio.mori@unifg.it; 3 Department of Biomedical Science and Human Oncology, Paediatric Unit, University of Bari, 70100 Bari, Italy; mariafelicia.faienza@uniba.it

Bone is an active tissue that remodels continuously throughout life [1]. It is a process involving different types of cells: osteoblasts, the bone-forming cells; the osteoclasts, the bone-resorbing cells; and the osteocytes, the mechanosensory cells. Their activity is strictly coordinated to preserve bone health [1,2]. In detail, the old or damaged bone is resorbed by osteoclasts and substituted with new bone matrix produced by osteoblasts. Osteoclasts arise from the fusion of monocyte-macrophage lineage cells and degrade bone matrix through the secretion of proteolytic enzymes and acid [3,4]. Osteoblasts differentiate from mesenchymal stem cells following the sequential activation of transcriptional factors and terminally become osteocytes [5,6]. Osteoblasts produce all the components of bone matrix; initially, it is secreted as unmineralized osteoid and over time mineralized by the deposit of hydroxyapatite [7]. 

The equilibrium between bone formation and resorption is tightly regulated without alterations in net bone mass or mechanical strength in physiological conditions [1,2]. However, dysregulation of this equilibrium determines the anomalous bone remodeling, leading to bone diseases [8,9]. This equilibrium moves in favor of osteoclasts in osteolytic diseases [9]. 

In this special issue, different authors reported the mechanisms regulating osteoclastogenesis in order to identify new therapeutic targets and to explore the pathogenesis of bone diseases. 

Osteoclasts differentiate thanks to the activity of two cytokines, the macrophage-colony stimulating factor (M-CSF) and the receptor activator of nuclear factor-κB ligand (RANKL) [3]. RANKL is expressed on osteoblasts and stromal cells; recently, it has also been reported that osteocytes are the major source of the cytokine. As reviewed by Kitaura et al. TNF-α directly increases RANKL levels in osteocytes [10]. Moreover, TNF-α positively affects sclerostin expression in osteocytes, which indirectly also augments the differentiation of osteoclasts. Thus, the osteocyte represents the master manager of bone resorption and effector in osteoclastogenesis.

Chen et al. demonstrated that N-[2-(4-acetyl-1-piperazinyl)-phenyl]-2-(2-chlorophenoxy) acetamide (PPOA-N-Ac-2-Cl) inhibited osteoclastogenesis in a dose-dependent manner, without affecting cell viability. PPOA-N-Ac-2-Cl modulated the expression of osteoclast markers, such as TRAF6, c-fos, DC-STAMP, NFATc1, MMP9, CtsK, and TRAP [11]. Consequently, F-actin ring formation and bone resorption decreased in osteoclast cultures treated with PPOA-N-Ac-2-Cl. Thus, Chen et al. suggested that PPOA-N-Ac-2-Cl may represent a potential therapeutic agent for the management of osteoclast-mediated bone diseases.

Interleukin (IL)-35 suppresses the inflammatory immune response and inhibits osteoclastogenesis [12,13]. Kamiya et al. investigated the synergistic effect of IL-35 and RANKL on osteoclastogenesis [14]. Co-stimulation of RAW cells with RANKL and IL-35 stimulated osteoclastogenesis significantly compared with RANKL treatment alone. Phosphorylations of ERK and p38 augmented following the simultaneous treatment with RANKL and IL-35 compared with RANKL or IL-35 alone. Consistently, the osteoclast formation induced by RANKL and IL-35 was inhibited by neutralization of ERK. In this study, IL-35 and RANKL induced osteoclastogenesis synergistically. Thus, considering the previous reports, IL-35 might play dual roles of destruction and protection in osteoclastogenesis according to the presence of RANKL.

Interleukin (IL)-33 is a member of the IL-1 family and it is known to inhibit osteoclastogenesis and bone resorption [15]. Ohori et al. evaluated the role of IL-33 on TNF-α-induced osteoclast formation and activity, demonstrating that the number and the activity of TRAP-positive cells induced by TNF-α were significantly reduced by the cytokine in vitro and in vivo [16]. IL-33 reduced IκB phosphorylation and NF-κB nuclear translocation. These results suggest that IL-33 inhibited TNF-α-induced osteoclastogenesis and bone resorption.

Another cytokine with pro-osteoclastogenic properties is LIGHT/TNFSF14 [17,18]. It is produced by immune cells, and interestingly regulates bone remodeling in basal and pathological conditions [17,18,19,20,21,22], but interestingly it also regulates the homeostasis of adipose tissue [23]. It is known that obese children have low bone mineral density and a greater risk of osteoporosis and fractures [24]. In the attempt to analyze the pathogenesis of this disease, Brunetti et al. demonstrated high levels of LIGHT in sera and on circulating cells of obese children and adolescents respect the controls [25]. The same authors also reported that in cultures of peripheral blood mononuclear cells from obese subjects, the addition of anti-LIGHT antibodies induced a significant osteoclastogenesis inhibition. Interestingly, the serum levels of LIGHT correlated with reduced bone mineral density and the grade of obesity, thus representing a valid therapeutic target to counteract both bone disease and obesity.

Skeletal abnormalities are also typical of gastrointestinal diseases that are characterized by anomalous immune cell activity and high levels of inflammatory cytokines in the bone marrow milieu, due to disturbed gut immune response, as reviewed by Ke et al. [26]. Gastrointestinal disease is known as an immune failure driven by numerous factors, including signaling molecules and cytokines. However, the mechanisms leading to bone loss in gastrointestinal diseases require further investigations. Ke et al. in their review discuss the key risk factors possibly contributing to intestinal disease-associated bone loss, and recapitulate current animal models, useful to bridge the gap between skeletal disease and intestinal inflammation [26]. In conclusion, this Special Issue on “Molecular Mechanisms Regulating Osteoclastogenesis” provides an overview of the mechanisms regulating osteoclastogenesis in physiological and pathological conditions, thus suggesting new potential therapeutic targets against bone diseases.
